# Development of eco-friendly antifungal and antibacterial adhesive derived from modified cassava starch waste/polyvinyl alcohol containing green synthesized nano-silver

**DOI:** 10.1038/s41598-023-40305-3

**Published:** 2023-08-16

**Authors:** Chaloton Jarensungnen, Kaewta Jetsrisuparb, Supranee Phanthanawiboon, Somnuk Theerakulpisut, Salim Hiziroglu, Jesper Theodorus Nicolaas Knijnenburg, Manunya Okhawilai, Pornnapa Kasemsiri

**Affiliations:** 1 Department of Chemical Engineering, Faculty of Engineering, Khon Kaen University, Khon Kaen, 40002 Thailand; 2 Department of Microbiology, Faculty of Medicine, Khon Kaen University, Khon Kaen, 40002 Thailand; 3 Energy Management and Conservation Office, Faculty of Engineering, Khon Kaen University, Khon Kaen, 40002 Thailand; 4 Department of Natural Resource Ecology and Management, Oklahoma State University, Stillwater, OK 74078 USA; 5 International College, Khon Kaen University, Khon Kaen, 40002 Thailand; 6 Center of Excellence in Responsive Wearable Materials, Chulalongkorn University, Bangkok, 10330 Thailand; 7 Metallurgy and Materials Science Research Institute, Chulalongkorn University, Bangkok, 10330 Thailand

**Keywords:** Materials science, Nanoscience and technology

## Abstract

Environmentally friendly biopolymer-based wood adhesives are an inevitable trend of wood product development to replace the use of harmful formaldehyde-based adhesives. In this research, a new eco-friendly modified cassava starch waste-based adhesive via carboxymethylation (CMS), and blending with polyvinyl alcohol (PVA), tannic acid (TA) and green synthesized silver nanoparticles (AgNPs) was prepared. The effects of TA content on green synthesis of AgNPs (Ag-TA) and bio-adhesive nanocomposite properties were investigated. The use of 5 wt% TA for AgNPs synthesis (Ag-TA-5) resulted in a uniform particle size distribution. The plywood prepared with Ag-TA-5 provided the highest dry and wet shear strength at 1.95 ± 0.11 MPa and 1.38 ± 0.3 MPa, respectively. The water absorption and thickness swelling of this plywood remarkably decreased up to 10.99% and 6.79%, respectively. More importantly, the presence of Ag-TA in CMS/PVA adhesive successfully inhibited the invasion of mold and bacteria. Based on the cyclic delamination test, the adhesive bond durability of bio-adhesive containing Ag-TA-5 could meet the requirement of the AITC Test T110-2007 and was comparable to commercial adhesives. The added advantage of the prepared bio-adhesive was its synthesis from agro-waste products and possible economically viable production at industrial level.

## Introduction

Aldehyde synthetic resins have been widely used as adhesives in various applications, especially in the field of wood-based materials due to their strong adhesion to many surfaces^1^. However, these resins still have the main drawback of formaldehyde emission. According to the World Health Organization (WHO), formaldehyde is categorized as a carcinogen that causes environmental and health problems, and consequently the manufacturing of environmentally friendly adhesives to replace aldehyde-based adhesives has raised significant attention^2^. Biopolymer-based adhesives are promising non-toxic, inexpensive, and renewable alternatives^3,4^. Recently, bio-adhesives from soy protein^5^, chitosan^6^, and carboxymethyl starch^7^ have been extensively studied, while the use of their by-products and wastes for advanced or even similar applications is rather limited^8^. The production of cassava starch is an important food industry. During starch manufacturing, the washing and extraction process produces large amounts of cassava starch waste (CSW) that is rich in starch and fiber^9^. Ray and Moorthy^10^ found that CSW contained as much as 50–70% starch, suggesting that it could be used as a potential binder. However, use of the natural adhesives is limited because of their poor physical and mechanical properties and modification is necessary to achieve the targeted properties^11^. Carboxymethyl starch (CMS) is a starch that is modified by etherification with carboxymethyl groups. The abundant polar carboxyl groups of CMS enhance the cohesive force of cellulosic materials^12^. Lamaming et al.^7^ prepared a bio-adhesive using CMS and polyvinyl alcohol (PVA). The use of CMS provided a good shear strength in the range of 0.29–0.54 MPa which was higher than the required standard for particleboard. Blending of CMS with PVA could further enhance the internal bond strength up to 86%, and the water absorption of the particleboard was reduced from 208.38% for CMS to 95.16% for CMS/PVA. Therefore, modification of CSW by carboxymethylation will improve its performance in adhesive applications.

Carbohydrate polymers that are used in the preparation of bio-adhesives may be easily eroded by fungi and bacteria, resulting in a negative effect on shortened life and low bonding durability of the adhesive^13^. Hence, the incorporation of antibacterial and antifungal agents into bio-adhesives could be an approach to overcome these problems^14^. Silver nanoparticles (AgNPs) have excellent antimicrobial properties and are widely used in biomedical and food packaging applications^4,15^. Huang et al.^16^ developed soy protein-based adhesives containing green synthesized AgNPs that could inhibit the growth of *Staphylococcus aureus* (*S. aureus*) and *Escherichia coli* (*E. coli*) and had long-term anti-mold properties. Among various techniques for AgNPs production, green synthesis is preferred because of the low energy consumption and environmental friendliness^17^. Several plants and plant extracts containing terpenoids, glycosides, alkaloids or phenolics can be used as bio-reducing agents to convert silver ions into AgNPs^18^. Tannic acid (TA) is a natural phenolic compound that has been used as a bio-reducing agent for synthesis of AgNPs^19^ and can also be used as a crosslinker or stabilizer for several kinds of biopolymers^20^. Hassanien and Khatoon^21^ found that green synthesized AgNPs using TA have good activity against bacteria and fungi. Ounkaew et al.^20^ demonstrated that green synthesized AgNPs with a suitable content of TA provided a good nanoparticle dispersion in the polymer matrix with enhanced mechanical properties and antibacterial activity. Moreover, Li et al.^22^ studied soy protein adhesives modified by chitosan/TA/AgNPs nanocomposites. The presence of TA and AgNPs in the adhesives enhanced dry and wet shear strength up to 171% and 93%, respectively. Based on literature, the use of green synthesized AgNPs as an antimicrobial agent for bio-adhesive would enhance its overall performance.

According to the literature, CMS/PVA/AgNPs nanocomposites have been developed for various applications. However, there is only little information on the synthesis of CMS from CWS and the use of green synthesized AgNPs by TA as reinforcing agent and crosslinker. Therefore, this research focused on the development of a new bio-adhesive based on modified CSW/PVA containing green synthesized AgNPs. The effects of TA content on the synthesis of AgNPs and bio-adhesive properties of the composites were studied. The performance of the bio-adhesives was measured for its antibacterial and antifungal properties water resistance and cost analysis.

## Experimental work

### Materials

Cassava starch waste (CSW) containing 29.86% amylose and 70.14% amylopectin was supplied by Kaen Charoen Co., Ltd, Khon Kaen, Thailand. Polyvinyl alcohol (average molecular weight of 1700–1800), sodium hydroxide (NaOH), ethanol, and silver nitrate (AgNO_3_) were obtained from RCI Labscan Limited, Bangkok, Thailand. Sodium mono chloroacetate (SMCA) with 98% purity and TA with molecular weight of 1701.2 g × mol^−1^ were purchased from Acros Organics and Sigma-Aldrich, Singapore, respectively. Isopropyl alcohol was obtained from Sigma-Aldrich, Singapore.

### Preparation of CMS

The CMS was prepared following Ounkaew et al.^23^ with slight modification. In brief, the dried CSW (40 g) was dispersed in 324 mL of isopropanol. A 30 mM NaOH solution was slowly added until the pH was approximately 11. The mixture was continuously stirred at 40 °C for 30 min. Next, SMCA (28.76 g) was added and stirred for another 1 h. The starch slurry was filtered and dispersed in 95% ethanol. The starch solution was mixed with 5 mL 38% HCl and then filtered and washed with 85% ethanol until the pH reached 7. Finally, the filtered starch cake was dried in an oven at 40 °C for 12 h to obtain the CMS.

### Synthesis of AgNPs

The AgNPs were synthesized by using TA as a reducing agent. First, TA solutions with different concentrations (1–7% w/v) were prepared with pH adjusted to 8 using 0.5 M Na_2_CO_3_. The TA solution was heated to 60 °C and a solution of 100 mM AgNO_3_ (0.6–10 mL of TA) was added. The mixture was stirred at 60 °C for 12 h under reflux to obtain the TA/AgNPs solution.

### Preparation of bio-adhesives

The bio-adhesives were prepared by mixing CMS/PVA as a polymer matrix by dissolving PVA (5 g) in 40 mL of deionized water at 90 °C. The CMS (5 g) was slowly added to the PVA solution while stirring at 60 °C until homogeneous. Next, 5 mL of as-synthesized TA/AgNPs solution was added to the CMS/PVA solution and stirred for 30 min at 60 °C to obtain the bio-adhesives. The CMS/PVA adhesives containing AgNPs and 1%, 3%, 5% and 7% TA were defined as Ag-TA-1, Ag-TA-3, Ag-TA-5 and Ag-TA-7, respectively.

### Characterization of bio-adhesives

The as-synthesized AgNPs were characterized using an UV–Vis spectrophotometer (Perkin-Elmer, Lambda 35, spectrometer, United States) at 300–550 nm.

X-ray diffraction of the samples was carried out using a SmartLab X-ray diffractometer (Rigaku, Japan) over a diffraction angle range of 2*θ* = 30°–70°; the diffractometer was equipped with a Cu Kα radiation source (wavelength l = 1.542 A°). A scan rate of 10° (2*θ*) at 40 kV and 30 mA was applied for the test. The average crystal size of the AgNPs was calculated by using Scherrer equation as in Eq. (1)

1 $$ Crystallite \, size \, = \, \frac{k\lambda }{{\beta \cos \theta }} $$

where k is crystallite shape factor of which a good approximation is 0.9, $\uplambda$ is the X-ray wavelength (nm), $\beta$ is the full width at half the maximum (FWHM) in radians of the X-ray diffraction peak, and $\theta$ is the Bragg angle.

The morphology of the bio-adhesives containing AgNPs was observed using transmission electron microscopy (TEM, JEM-2100 Plus, JEOL, Japan). The samples were dropped on 400-mesh carbon-coated Cu grids (one drop for each solution) and dried at room temperature for 24 h prior to analysis.

The functional groups of the bio-adhesives containing AgNPs were analyzed by attenuated total reflection infrared (ATR-FTIR) spectroscopy (Jasco 4200, Tokyo, Japan). The FT-IR spectra were collected in the range of 4000–550 cm^−1^ with 64 scans at a resolution of 4 cm^−1^.

**Figure 1:**
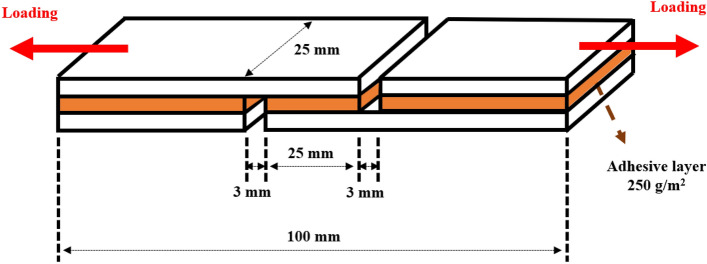
Plywood specimen for shear strength test.

The shear strength was tested according to the industry standard GB/T 9846–2015. The bio-adhesive was spread on one side of a wood specimen at a coating density of 250 g × m^−2^, and then compressed at 1.0 MPa for 1 h at 40 °C, as shown in Fig. 1. The obtained plywood specimen was stored at 23 ± 2 °C and 50 ± 5% relative humidity (RH) for 48 h before testing. The shear strength of the plywood was measured using a Universal testing machine (UTM, Shimadzu, Model: EHF-EG10-20L, Japan) under dry and wet conditions. For the wet conditions, the sample was immersed in water at 27 ± 3 °C for 3 h and dried at 40 °C for 20 min, according to the procedure reported previously^24^. The bonding area was 25 × 25 mm. The test was performed with a shear rate of 10 mm × min^−1^ at room temperature.

Six 25 × 25 mm plywood samples were used to test water adsorption and thickness swelling for each adhesive solution. The initial weight and thickness of the samples were measured after drying the samples in an oven at 40 °C to achieve a constant weight. Next, the samples were submerged in water. The increase in thickness and weight of each sample were recorded at pre-determined intervals (1, 2, 4, 6, 8, 10, 12, 24, 48 and 72 h). The water adsorption and thickness swelling were calculated following Eqs. (2) and (3), respectively.

2 $$ WA = \, 100 \times \frac{{W_{f} - W_{i} }}{{W_{i} }} $$

where WA is water adsorption (%), W_i_ is initial weight of sample (g) and W_f_ is weight of sample after immersing in water at pre-determined intervals (g).

3 $$ TS = \, 100 \times \frac{{T_{f} - T_{i} }}{{T_{i} }} $$

where TS is thickness swelling (%), T_i_ is initial thickness of sample (mm) and T_f_ is thickness of sample after immersing in water at pre-determined intervals (mm).

The antibacterial activities of the bio-adhesive samples were tested using the agar diffusion method on Gram-negative *Escherichia coli* (*E. coli*, ATCC25922) and Gram- positive *Staphylococcus aureus* (*S. aureus,* ATCC25923) bacteria. The bacteria were cultured in brain heart fusion broth at 37 °C. The bacterial culture was diluted to achieve a turbidity of approximately 10^8^ CFU mL^−1^. Bacteria were seeded on Muller Hinton Agar plates by the swab plate technique. The inhibition zone (mm) was measured after 12 h incubation at 37 °C.

For the antifungal test, *Penicillium *sp. was the selected mold according to the Chinese national standard (GB/T 18261-2013) and the test performed followed the swab plate technique. Briefly, *Penicillium *sp. was inoculated in potato dextrose broth under sterile conditions and cultivated in a shaking incubator at 28 °C and 85% RH for 2 h. The cultured broth was applied on the surface of potato dextrose agar by cotton swab for 3 panels. The bio-adhesive samples were placed on the cultured test molds in petri dishes and incubated at 28 ± 2 °C and 85% RH for 72 h. The changes in surface morphology of samples were recorded by digital camera.

The cyclic delamination test was used to evaluate the durability of adhesive bonds in plywood. This test was performed according to AITC Test T110-2007 standard. A sample with dimensions 80 × 80 mm was placed into a pressure chamber with the end grain surfaces freely exposed to water at a temperature of 25 ± 3 °C. A vacuum condition of 85 kPa was applied on the samples for 30 min followed by a pressure of 510 kPa for 2 h. The sample was then taken out and dried at 71 ± 3 °C until the weight was 12–15% of the initial weight. The obtained sample was used to determine the bond line evaluation using Eq. (4)

4 $$ D = \, 100 \times \frac{{L_{D} }}{{L_{G} }} $$

where D is the delamination value (%), L_D_ is the total delamination length (mm) for all 4 surfaces and L_G_ is the perimeter of all bond lines in the test block (mm).

## Results and discussion

### Structural characterization of green synthesized AgNPs and bio-adhesive nanocomposites

**Figure 2:**
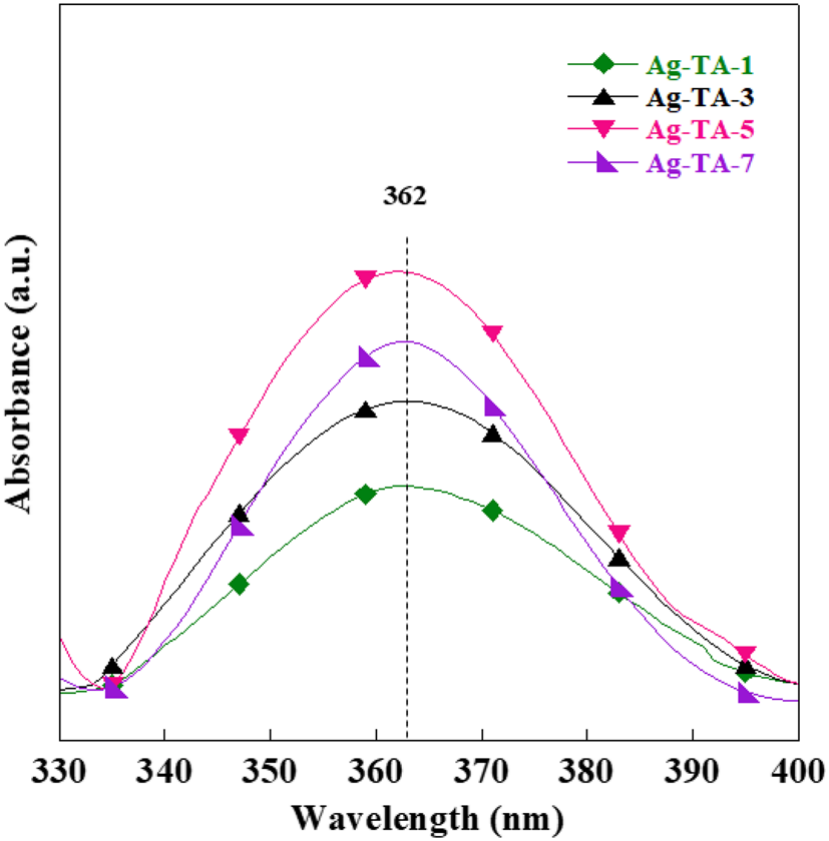
Absorbance spectra of green synthesized AgNPs at various concentrations of TA.

The absorbance spectra of green synthesized AgNPs at various concentrations of TA are illustrated in Fig. 2. The position and intensity of the absorption peak of AgNPs can be found in the range of 350–430 nm depending on size, shape, and surface capping agents^25–27^. The absorption peak of green synthesized AgNPs with TA was found at 362 nm. This peak intensity increased with increasing TA content from 1 to 5%, which was attributed to the formation of AgNPs at high concentration^28,29^. The peak intensity decreased when 7% TA was used, implying the aggregation of AgNPs. The improper thickness of reducing agent layer around AgNPs might decrease the electrostatic repulsion between AgNPs leading to aggregation^27,30^.

**Figure 3:**
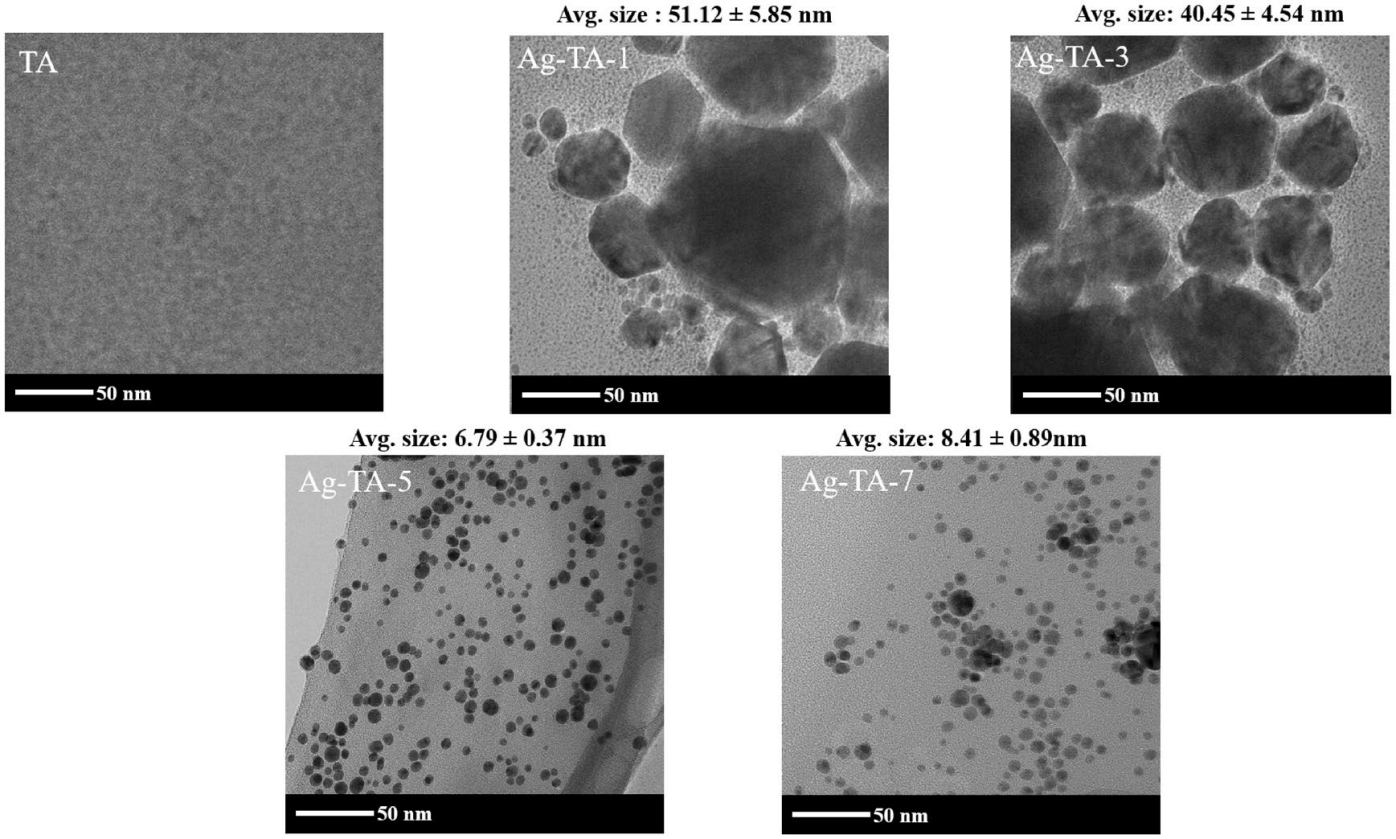
TEM images of green synthesized Ag-TA at various concentrations of TA.

The obtained UV–Vis spectral results were in good agreement with the TEM analysis. Figure 3 shows the effects of TA on the particle size and distribution of AgNPs. An increase in TA content from 1 to 5 wt% lead to smaller average particle size of AgNPs from 51.12 ± 5.85 nm (Ag-TA-1), 40.45 ± 4.54 nm (Ag-TA-3) to 6.79 ± 0.37 nm (Ag-TA-5), where a further increase in TA content to 7% result in aggregation of AgNPs with larger particle size (8.41 ± 0.89 nm). Uniform distribution of AgNPs was obtained at 5 wt% TA contents. Hence, it was concluded that the use of 5 wt% TA was optimal for AgNPs synthesis. The use of a bio-reducing agent consisting of flavonoids and polyphenols at its optimal concentration can produce negative charges and the proper layer thickness of reducing agent around AgNPs to achieve a uniform distribution of AgNPs with small particle size^31,32^. A similar observation was reported by Zhang et al.^33^: when increasing the TA content (from AgNO_3_:TA molar ratio of 1:0.1–1:0.5), the AgNPs particle size became smaller with uniform distribution. However, further increasing the TA content (AgNO_3_:TA ratio of 1:0.75) increased the AgNPs particle size due to a lower AgNO_3_ concentration, resulting in a lower reaction rate.

**Figure 4:**
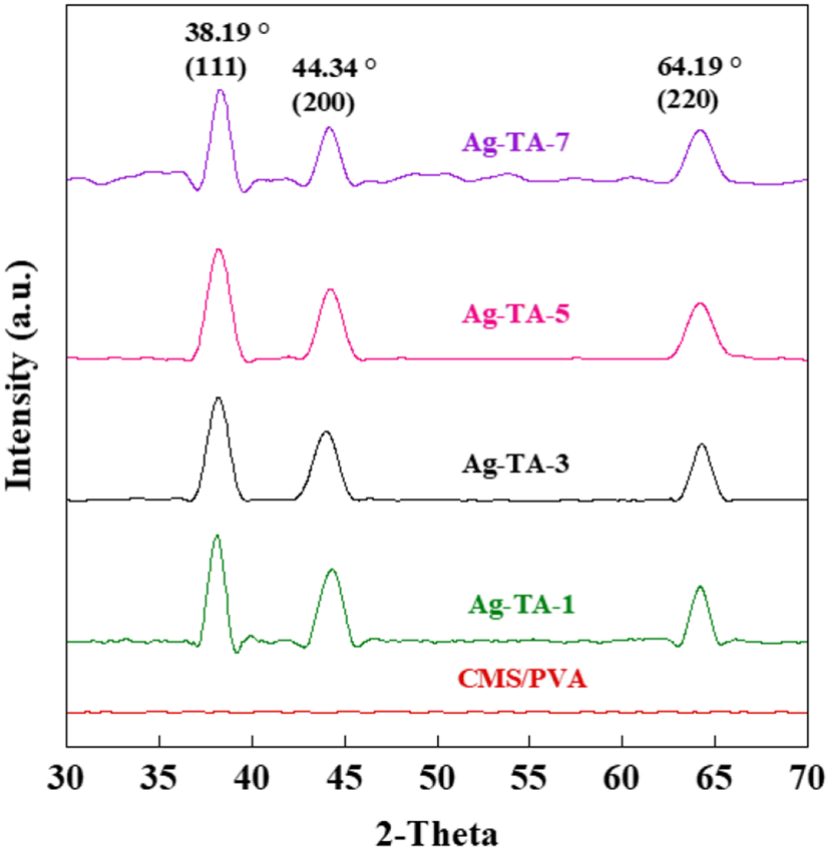
XRD patterns of Ag-TA at different concentrations of TA.

The XRD patterns of the bio-adhesives at different concentrations of TA are presented in Fig. 4. Diffraction peaks were observed at 2θ values of 38.19, 44.34 and 64.19° which corresponded to the crystallographic planes of silver at 111, 200 and 220, respectively. This confirmed the face-centered-cubic (FCC) structure of silver according to the Joint Committee of Powder Diffraction Standard (JCPDS, No. 087-0720/No. 4-0783)^34^. In addition, broader peaks were observed in the following order: Ag-TA-5 > Ag-TA-7 > Ag-TA-3 > Ag-TA-1. Generally, broader peaks in the XRD patterns are attributed to smaller crystallite sizes^35,36^. By using the Scherrer equation (Eq. 1), the average crystallite size of Ag-TA-1, Ag-TA-3, Ag-TA-5 and Ag-TA-7 was 7.47, 6.88, 6.11 and 6.60 nm, respectively. The Ag-TA-5 had a smaller crystalline size when compared to other samples, which further confirmed the appropriate TA content for green synthesis of AgNPs.

**Figure 5:**
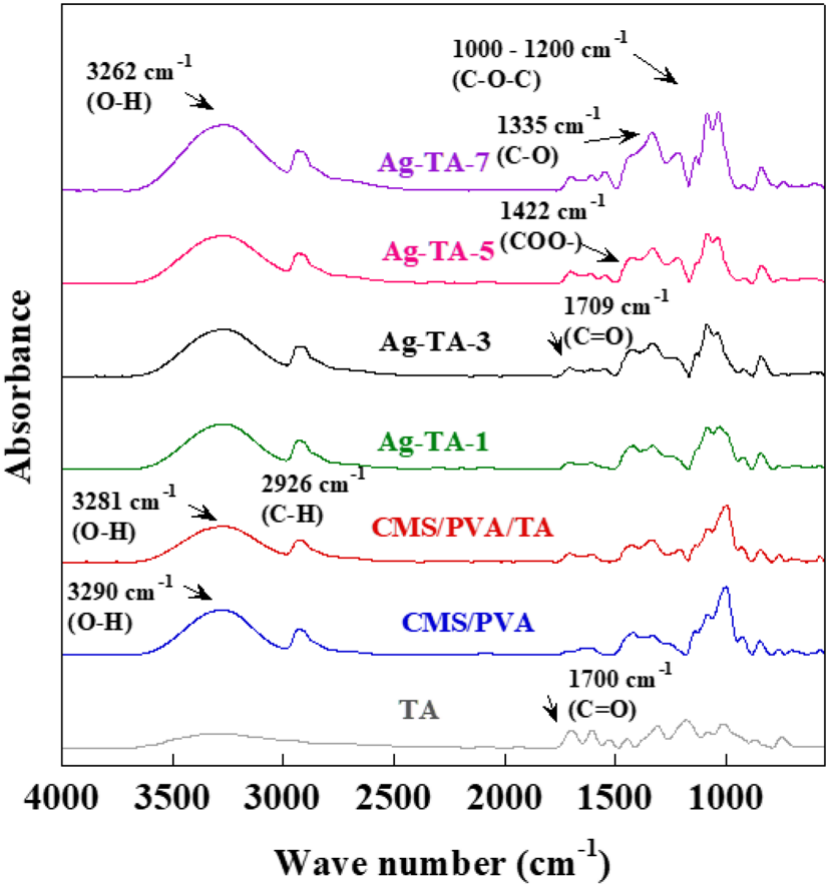
FT-IR spectra of the blend of CMS/PVA, CMS/PVA crosslinked with TA and Ag-TA-5.

FT-IR spectra of the blended CMS/PVA, CMS/PVA crosslinked with TA and Ag-TA-5 are illustrated in Fig. 5. For TA, a broad band in the range of 3000–3500 cm^−1^ was attributed to OH stretching vibrations. The band at 1700 cm^−1^ indicated the presence of carbonyl groups (C=O) whereas the presence of aromatic C=C bonds was confirmed by peaks at 1604 cm^−1^ and 1527 cm^−1^. The band at 1297 cm^−1^ was assigned to C–H vibrations and the band in range of 1125–1316 cm^−1^ corresponded to C–O and C–H vibrations^20,37^. The blend of CMS/PVA showed the characteristic peaks of both CMS and PVA. The bands at 3290 cm^−1^ and 2926 cm^−1^ were attributed to OH stretching and C–H stretching vibrations of CH_2_ groups, respectively. The intense bands at 1594 cm^−1^ and 1431 cm^−1^ were assigned to C=O stretching vibrations. The characteristic bands around 880–1200 cm^−1^ corresponded to C–O stretching vibrations^38,39^. When the blend of CMS and PVA was mixed with TA, a new peak at 1738 cm^−1^ was found for CMS/PVA/TA and assigned to the ester group (C=O) of TA^40^. Furthermore, a peak shift from 3290 to 3281 cm^−1^ was also observed. This shift to lower wavenumbers indicated the formation of intra- or intermolecular hydrogen bonds between TA and the CMS/PVA blend^41^. The presence of AgNPs in the bio-adhesive showed a peak shift from 3281 to 3262 cm^−1^, as shown in the spectrum of Ag-TA-5 due to the coordination of electron-rich oxygen groups of AgNPs with O–H groups in the polymer matrix^42^.

### Gluability of bio-adhesive nanocomposite

**Figure 6:**
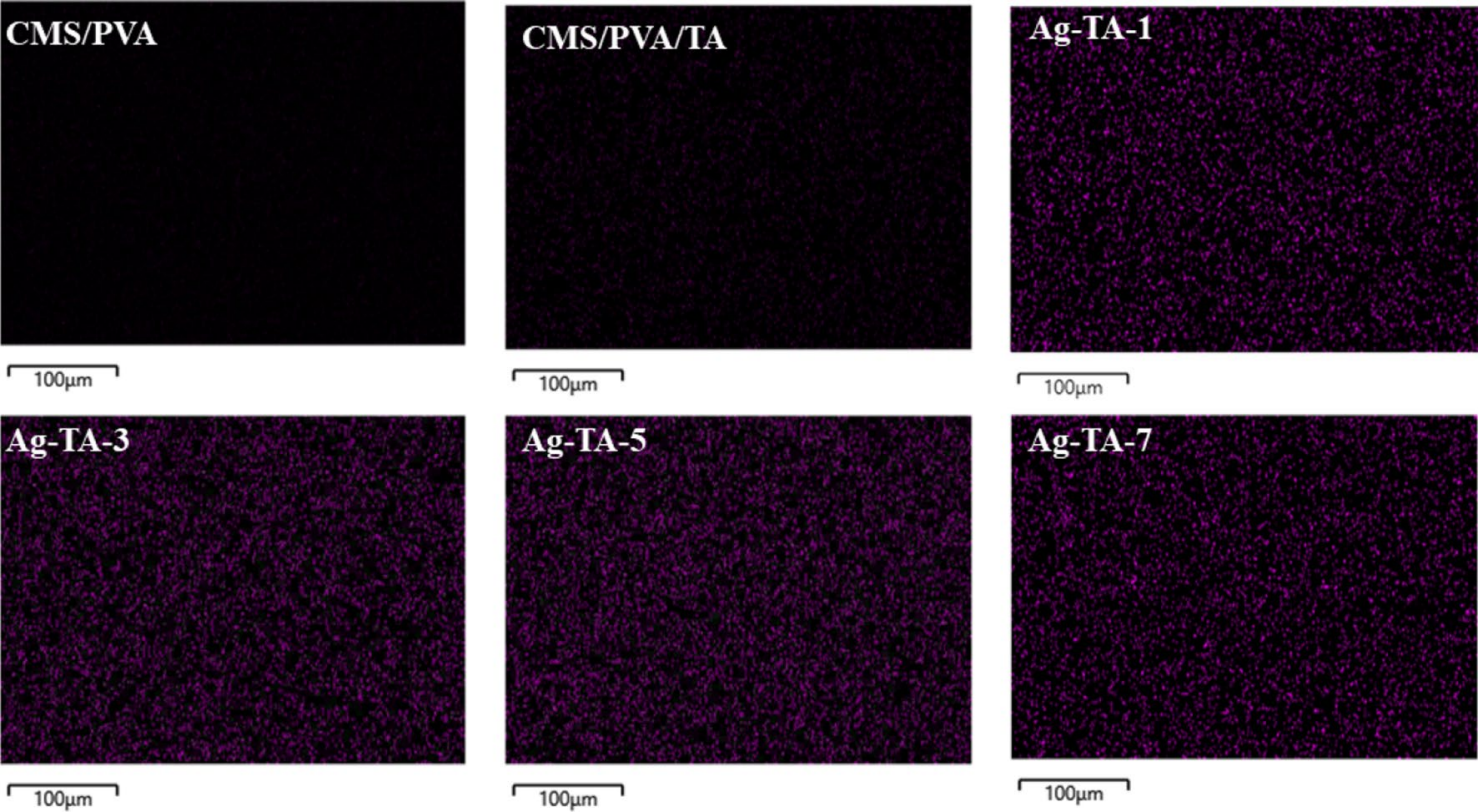
EDS mapping images of fracture surfaces.

**Figure 7:**
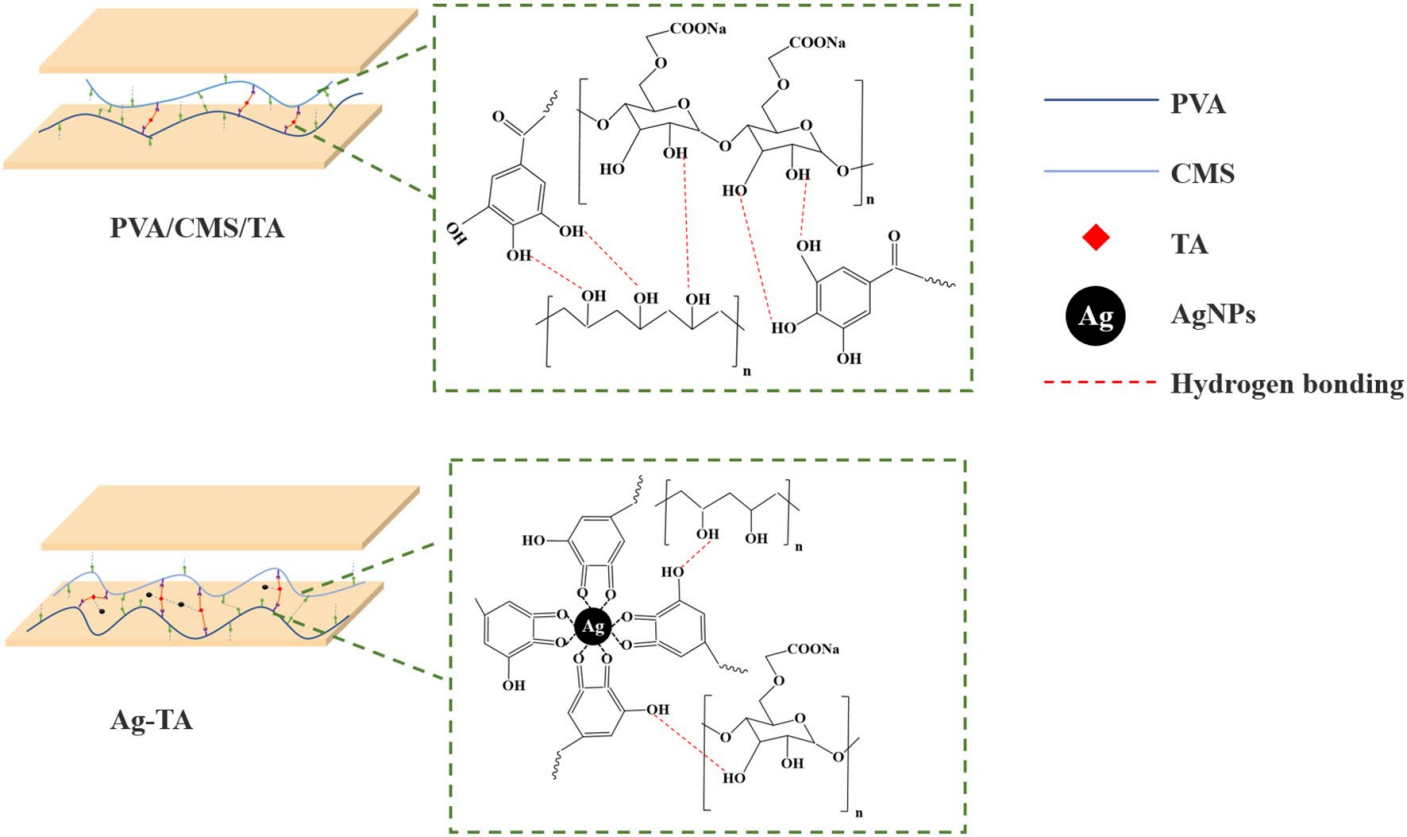
Possible adhesion mechanism of the CMS/PVA/TA and Ag-TA.

The gluability of bio-nanocomposite adhesive was measured in terms of the dry and wet shear strength, as shown in Table 1. The dry and wet shear strength of the CMS/PVA crosslinked with TA and bio-nanocomposite adhesives were substantially greater than that of CMS/PVA. The presence of TA as the crosslinker in CMC/PVA enhanced the cohesive strength for load-transfer ability^43,44^. When AgNPs were incorporated into the bio-adhesives, the shear strength increased from 0.57 ± 0.02 MPa (CMS/PVA) to 1.95 ± 0.11 MPa (Ag-TA-5). Xing et al.^45^ found that the starch adhesive reinforced with smaller nanoparticles provided a greater shear strength by improving the contact between molecular chains of adhesive and wood. Kasemsiri et al.^46^ suggested that the uniform distribution of filler in adhesive enhanced the surface roughness and interface by increasing the interaction area and the lap shear strength. As shown in Fig. 6, EDS mapping images of fracture surfaces of the adhesives revealed that Ag-TA-5 had more uniformly dispersed particles in the adhesive compared to other bio-adhesive samples. The aggregation of AgNPs particles was observed in Ag-TA-7. The poor dispersion of filler in the polymer matrix negatively affected the mechanical performance^47^. Furthermore, examination of the adhesive morphology on the fractured surface of wood by SEM analysis revealed the different characteristic surfaces related to mechanical properties (Figure S1). The CMS/PVA showed a loose adhesive layer indicating poor cohesion. The fractured surface of CMS/PVA/TA was smoother and more compact, which was attributed to a denser crosslinking within the adhesive layer^48–50^. For the adhesives incorporated with Ag-TA, rough fracture surfaces were found. This observation implied that the adhesive layer had a strong cohesion^48^, which was consistent with the obtained shear strength under dry and wet conditions. The possible adhesion mechanism of the CMS/PVA/TA and Ag-TA is presented in Fig. 7. The crosslinked CMS/PVA/TA via hydrogen bonds had smooth surface and compact structure, whereas the presence of Ag-TA in CMS/PVA/TA had a rougher adhesive layer which improved adhesion.

**Table 1:** Dry and wet shear strength of the bio-adhesive.

Sample	Dry shear strength (MPa)	Wet shear strength (MPa)
CMS/PVA	0.57 ± 0.02	0.32 ± 0.01
CMS/PVA/TA	1.28 ± 0.05	0.67 ± 0.04
Ag-TA-1	1.32 ± 0.19	0.74 ± 0.03
Ag-TA-3	1.56 ± 0.17	0.82 ± 0.07
Ag-TA-5	1.95 ± 0.11	1.38 ± 0.3
Ag-TA-7	1.62 ± 0.06	1.28 ± 0.05

When using Ag-TA adhesive in plywood, the wet shear strength of all Ag-TA samples was higher than 0.7 MPa which meets the requirements of the Chinese national standard (GB/T 9846-2015) for indoor plywood. In addition, the dry and wet shear strength of Ag-TA-5 was comparable to previously reported adhesives for plywood^5,24,48,51–56^, as summarized in Table 2. Based on the obtained results, the use of CMS from CSW combined with green synthesized AgNPs would create a new eco-environmentally friendly adhesive and add value to CSW.

**Table 2:** Dry and wet shear strength of adhesives for plywood.

Sample	Wet shear strength (MPa)	References
Tannin	0.49	^51^
Soy protein/acrylamide/ammonium persulfate incorporated with tannic acid–Fe^3+^	1.28	^52^
Soy protein isolate/bromelain/tannic acid-Zn^2+^	1.36	^5^
Starch-cellulosic adhesive	0.83	^24^
Waterborne-epoxy resin/soy protein/water-soluble polyacrylamide	0.73	^53^
Blood meal-based bio-adhesive/triglycidylamine (TMB)	1.27	^54^
Modified Soymeal adhesive/modified nanocrystalline cellulose	1.08	^55^
Soybean meal flour/sodium dodecyl sulfate/1,4-butanediol diglycidyl ether	1.36	^56^
Soy protein/ acrylic acid/calcium sulfoaluminate	1.21	^48^
Ag-TA-5	1.38 ± 0.3	This study

### Antibacterial and antifungal activities of bio-adhesive nanocomposite

**Figure 8:**
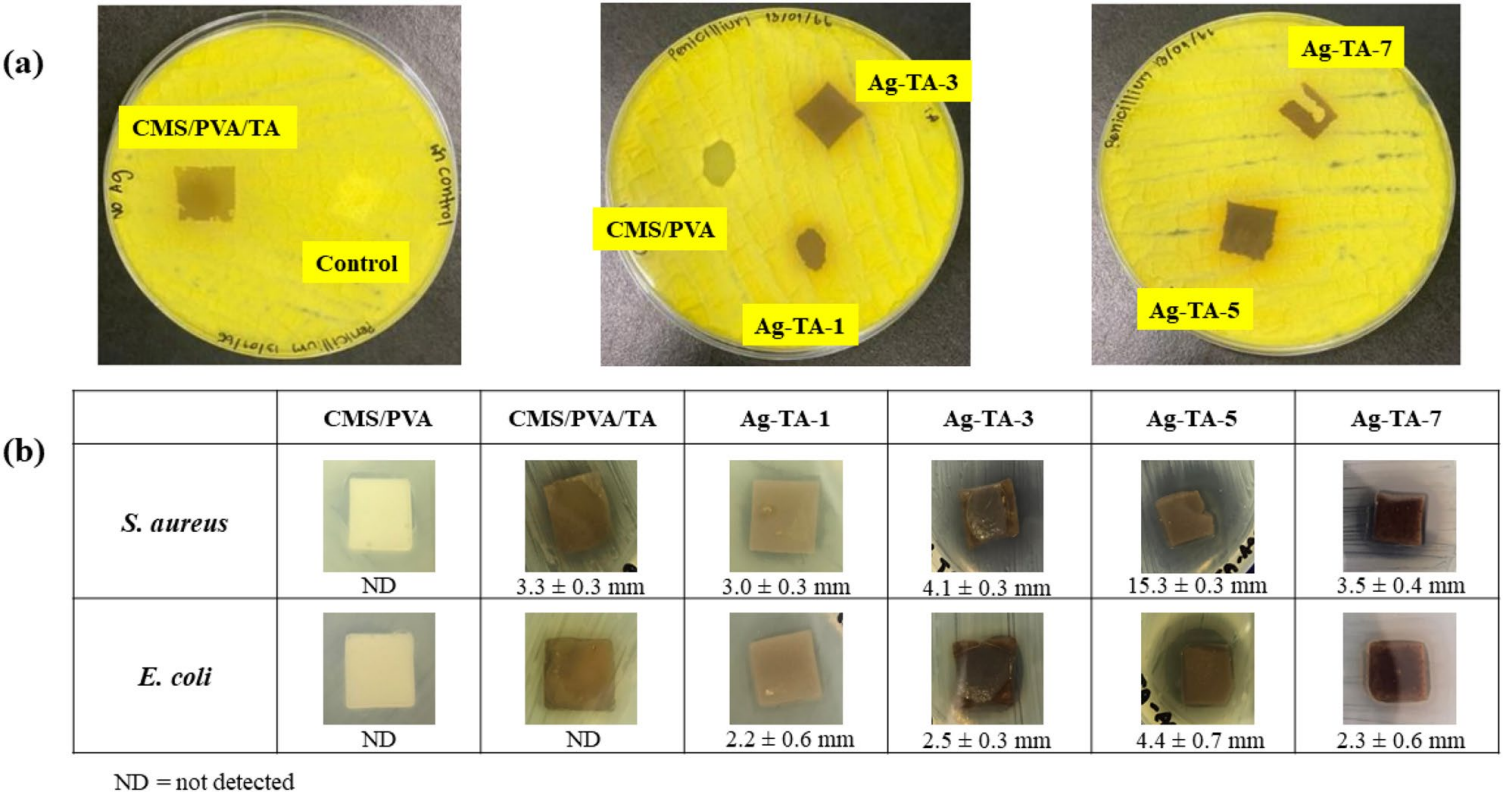
(**a**) Antifungal and (**b**) antibacterial activities of the bio-adhesive nanocomposites.

Most bio-adhesives derived from polysaccharides are easily infected by molds and bacteria. Thus, fungal and bacterial resistance is a necessary property for commercial application. Tests of antifungal and antibacterial activities of the cured or crosslinked adhesives were conducted to study the adhesive performance for its actual application^57–59^. Figure 8a,b show the antifungal and antibacterial activities of the bio-adhesive nanocomposites, respectively. It can be clearly seen in Fig. 8a that Ag-TA-3 and Ag-TA-5 showed a clean surface whereas other samples were covered with mold. This confirmed that Ag-TA-3 and Ag-TA-5 could inhibit the growth of *Penicillium *sp. The inhibitory zones for *S. aureus* and *E. coli* are shown in Fig. 8b. The CMS/PVA displayed no obvious inhibition whereas the CMS/PVA/TA had an inhibitory zone of 3.3 ± 0.3 mm against *S. aureus.* The largest inhibitory zones in the presence of both *S. aureus* and *E. coli* were found for Ag-TA-5, suggesting the highest antibacterial activity.

The antimicrobial activity of TA involves several actions such as hindered cell metabolism, increased permeability of the microbial cell membrane and deprivation of extracellular microbial enzyme^39,60,61^. Previous studies reported that the properties of AgNPs combined with TA produced synergistic antibacterial activities by increasing the permeability of the microbial cell membrane^20,62,63^. Furthermore, AgNPs also stimulate the generation of reactive oxygen species for attacking microbial cells and also interrupt the replication of bacterial DNA and the signal transduction pathways in the cells^64^.

### Water absorption of as-prepared plywood by bio-adhesive nanocomposite

**Figure 9:**
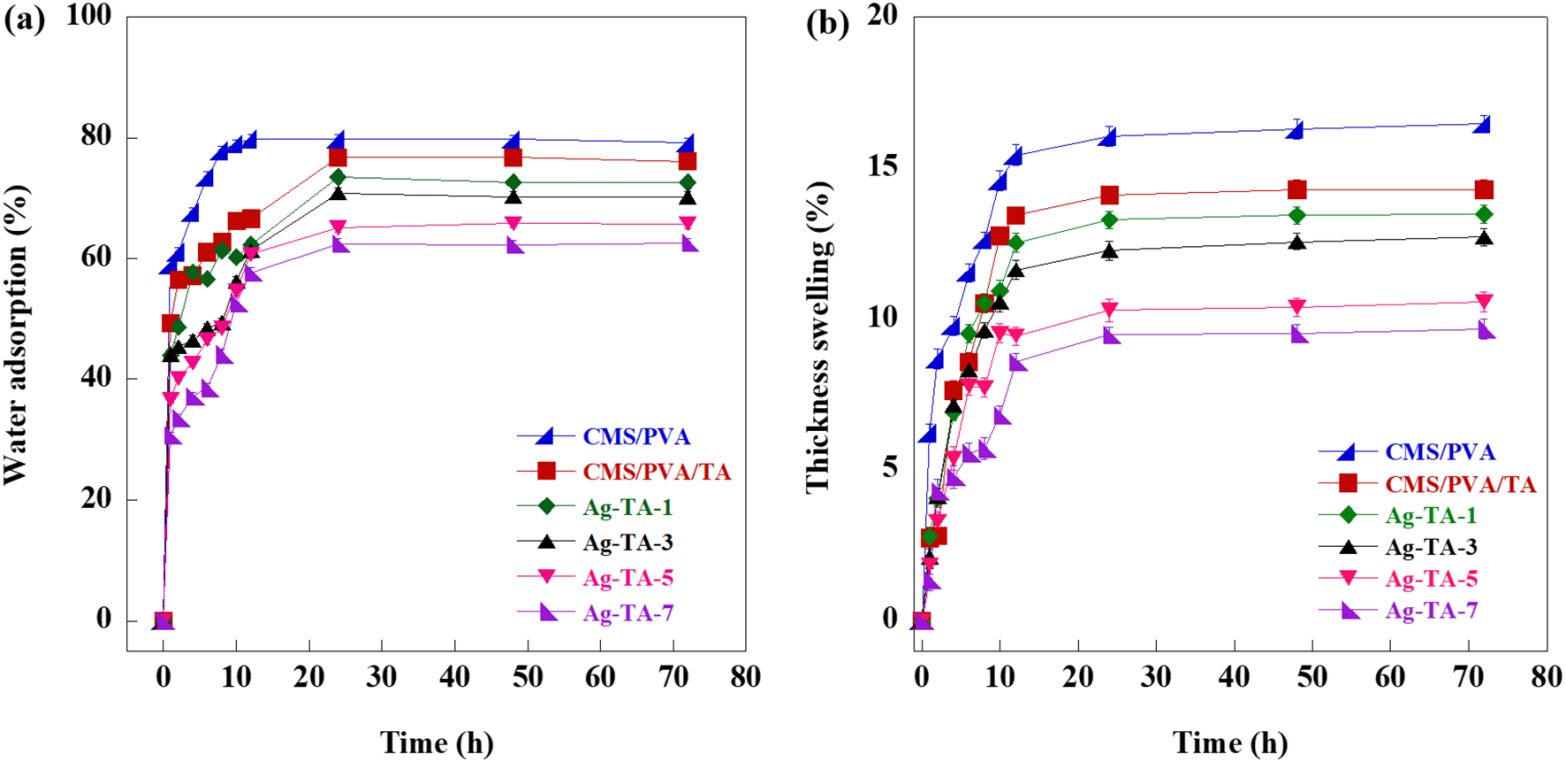
The percentages of (**a**) water absorption and (**b**) thickness swelling of the as-prepared plywood by Ag-TA adhesive.

One of the critical issues when using plywood is the change in dimensions in wet or highly humid conditions^65,66^. The water absorption of plywood panels could be explained by two main behaviors, namely (1) hydrogen bonding between the hydroxyl groups of water molecules and cellulosic cell wall of wood and (2) diffusion of water molecules into the interface between wood and adhesive^67^. The water uptake and thickness swelling of the as-prepared plywood by Ag-TA adhesive are shown in Fig. 9a,b. The water uptake and thickness swelling of the as-prepared plywood by Ag-TA after 72 h were 62.61–79.13% and 9.47–16.45%, respectively. The water resistance capability of the obtained plywood was comparable to plywood prepared with chitosan (65.7% water uptake and 15% thickness swelling)^67^ and conventional plywood (74.3% water uptake)^68^. The use of Ag-TA-7 in plywood preparation could decrease the water uptake by 10.99% and thickness swelling by 6.79%. This decrease in water uptake is due to the presence of TA which creates a crosslinking network and prevents the infiltration of water^43^. Generally, a low swelling percentage of crosslinked polymer reflects a high degree of crosslinking^69^. The crosslinking density of a polymer network is calculated by the Flory–Rehner equation as shown in Eqs. (S1–S2) and summarized in Table S1. The calculated crosslink density of the CMS/PVA and CMS/PVA/TA were 22.67 ± 1.51 g × cm^−3^ and 27.63 ± 1.84 g × cm^−3^, respectively. The incorporation of Ag-TA into PVA/CMS/TA increased the crosslink density from 45.48 ± 1.51 g × cm^−3^ for Ag-TA-1 to 64.09 ± 1.07 g × cm^−3^ for Ag-TA-7. The presence of TA and Ag-TA could create a tighter structural network resulting in a decreased swelling. In addition, the presence of nanofiller in the polymer matrix created tortuosity, resulting in a slower diffusion of water molecules^70^. Therefore, the incorporation of AgNPs into bio-adhesives could further enhance the water resistance of the plywood.

The water sorption kinetics of the plywood samples were studied to understand the water sorption mechanism and predict the water uptake behavior of the materials^71^. Fick's theory is widely used to analyze the diffusion mechanism and kinetics of water sorption. The generalized equation is expressed in Eq. (5)

5 $$ \frac{{M_{t} }}{{M_{\alpha } }} = kt^{n} $$

where M_t_ is the weight (g) at time t (s), M_α_ is the equilibrium weight (g), k and n are constants. The diffusion behavior can be classified as super case II (n > 1), case II (n = 1), anomalous (1/2 < n < 1), classical/Fickian (n = 1/2), or pseudo-Fickian (n < 1/2).

The n value can be calculated from the slope of the plot of log Mt/Mα versus log t*.* The values of n were 0.12–0.18, which implied pseudo-Fickian diffusion. Pritchard^72^ suggested that the early stage of diffusion for the pseudo-Fickian behavior was similar to Fickian diffusion, but at a later stage the rate of approaching equilibrium is delayed. This is possibly due to the influences of concurrent diffusion and sorption behavior. The obtained n values were similar to those found in the literature for other wood composites^73–75^.

The diffusion coefficient (D) is the most important parameter representing the ability of solvent molecules to penetrate inside the material structure. The D value can be calculated following Eq. (6)

6 $$ \frac{{M_{t} }}{{M_{\alpha } }} = \, 4\left[ {\frac{Dt}{{\pi L^{2} }}} \right]^{2} $$

where D is the diffusion coefficient (m^2^ × s^−1^), M_t_ is the weight (g) at time t (s), M_α_ is the equilibrium weight (g) and L is the thickness of the sample (mm).

The D values for plywoods were in the range of 0.78 × 10^–11^–4.17 × 10^–11^ m^2^ × s^−1^. The presence of Ag-TA-7 in the bio-adhesive decreased the water diffusion by up to 81.29%, which implied that the incorporation of AgNPs combined with TA in the adhesive remarkably enhanced hydrophobicity of the plywood.

### Thickness swelling of as-prepared plywood by bio-adhesive nanocomposite

Figure 9b shows the thickness swelling of the plywood samples with a remarkable increase during the first stage (0–10 h). The absorption of water in cellulose fiber and adhesive caused the thickness swelling of the plywood as suggested by Khalil et al.^76^ After 24 h, the thickness swelling values of the samples were 9.47–16.05%, which was lower than those of prepared plywood by glyoxalated lignin-urea-dialdehyde starch (48–63%)^77^ and in the same range as other plywoods prepared by phenol formaldehyde (6.67%), phenol formaldehyde/lignin (9–12%)^78^ and natural rubber latex/isocyanate crosslinked starch (13–20%)^66^. The lowest thickness swelling was found in the prepared plywood by Ag-TA-7 with improved dimensional stability of up to 9.47%. The densely crosslinked polymer network and compact structure of the nanocomposite adhesive hindered water penetration into the matrix, leading to a higher water resistance^43^.

### Durability of the adhesive bond cyclic delamination test

Based on the antifungal, antibacterial, and mechanical properties, material Ag-TA-5 presented the best performance with a water resistance capability that was slightly lower than Ag-TA-7. Therefore, Ag-TA-5 was selected to test the adhesive bond durability by cyclic delamination test under wet and dry conditions. As shown in Table 3, the delamination value of the plywoods was 7.56%, which meets the requirement (< 8%) of adhesive qualification for hardwood according to AITC Test T110-2007. This delamination value was lower than that of some adhesives such as epoxy, resorcinol, phenol resorcinol formaldehyde and emulsion polymer isocyanate, and comparable with polyurethane^79,80^ (Table 3).

**Table 3:** Comparison of delamination values of adhesives tested according to AITC Test T110-2007.

Adhesive sample	Wood species	Delamination (%)	References
Resorcinol	Hard wood (yellow birch)	30.9	^79^
Emulsion polymer isocyanate	Hard wood (eucalyptus)	15.7	^80^
Polyurethane	Hard wood (eucalyptus)	7.6	^80^
Epoxy	Hard wood (eucalyptus)	10	^80^
Phenol resorcinol formaldehyde	Hard wood (eucalyptus)	12	^80^
Ag-TA-5	Hard wood (teak)	7.56	This research

### Cost analysis for manufacturing

For industrial production of environmentally friendly adhesives, several requirements such as renewable raw material, cost, manufacturing techniques and product properties have attracted attention^24^. Here, the synthesis cost for the lab-scale manufacturing of 1000 mL of Ag-TA-5 were calculated (Table S2). Assumptions for the cost analysis were made according to Spiridon et al.^81^ with minor modifications. The raw material cost, energy consumption and operation expenditures were assumed to be the same for different locations, and all equipment purchasing costs and labor costs have not been included in this calculation. The manufacturing cost of Ag-TA-5 was 38.35 USD per 1000 mL. The obtained results would be beneficial for further development of environmentally friendly adhesive integrated antifungal and antibacterial properties.

## Conclusions

In this work, bio-based wood adhesives were successfully synthesized from CMS/PVA copolymer, TA, and Ag-TA. The TA acted as the crosslinking agent for the CMS/PVA copolymer and reducing agent for green synthesis of AgNPs. The use of TA at 5 wt% in AgNPs synthesis provided a good distribution of nanoparticles which enhanced the antifungal and antibacterial activities of the bio-nanocomposite adhesives. The dry and wet shear strengths of the prepared plywood bonded by Ag-TA-5 increased by 242% and 331%, respectively, when compared with the CMS/PVA adhesive. The prepared plywood by Ag-TA-5 showed a slight inferiority in terms of water resistance compared to the prepared sample by Ag-TA-7. The water resistance of plywood remarkably improved up to 10.99% for water absorption and 6.79% for thickness swelling when Ag-TA-5 was used. In addition, the durability of the adhesive bond of Ag-TA-5 could meet the requirement of AITC Test T110-2007 and was comparable to those of commercial adhesives. This study not only produced an environmentally friendly adhesive via a green process, but also introduced an effective approach for use of agricultural waste as a major material in wood adhesive manufacturing.

### Supplementary Information

Supplementary Information.

## Data Availability

The datasets used and/or analyzed during the current study are available from the corresponding author on reasonable request.
