# Supplementary material for: Development of eco-friendly antifungal and antibacterial adhesive derived from modified cassava starch waste/polyvinyl alcohol containing green synthesized nano-silver
Source: Sci Rep. 2023 Aug 16;13:13355. doi: 10.1038/s41598-023-40305-3 (PMC10432455; doi:10.1038/s41598-023-40305-3)
Supplement: Supplementary file 1 — Supplementary Information. [file 41598_2023_40305_MOESM1_ESM.doc]

**Development of eco-friendly antifungal and antibacterial adhesive derived from modified cassava starch waste/polyvinyl alcohol containing green synthesized nano-silver**

**Flory–Rehner equation**

$\nu=\text{ }-\frac{\ln(1-V_{p})+V_{p}+\chi V_{p}^{2}}{V_{0}d_{r}(V_{p}^{1/3}-\frac{V_{p}}{2})}$ Eq S1

when $\nu$ is crosslink density (g×cm^-3^)

*χ* is the polymer–solvent interaction parameter with value of 0.49

$V_{p}=\text{ }\frac{\frac{1}{d_{r}}}{\frac{Q_{m}}{d_{0}}+\frac{1}{d_{r}}}$ Eq S2

when $V_{p}$ is calculated from the mass swollen ratio ($Q_{m}$)

$d_{0}$and $d_{r}$ are the densities of the solvent and polymer (g×cm^-3^), respectively.


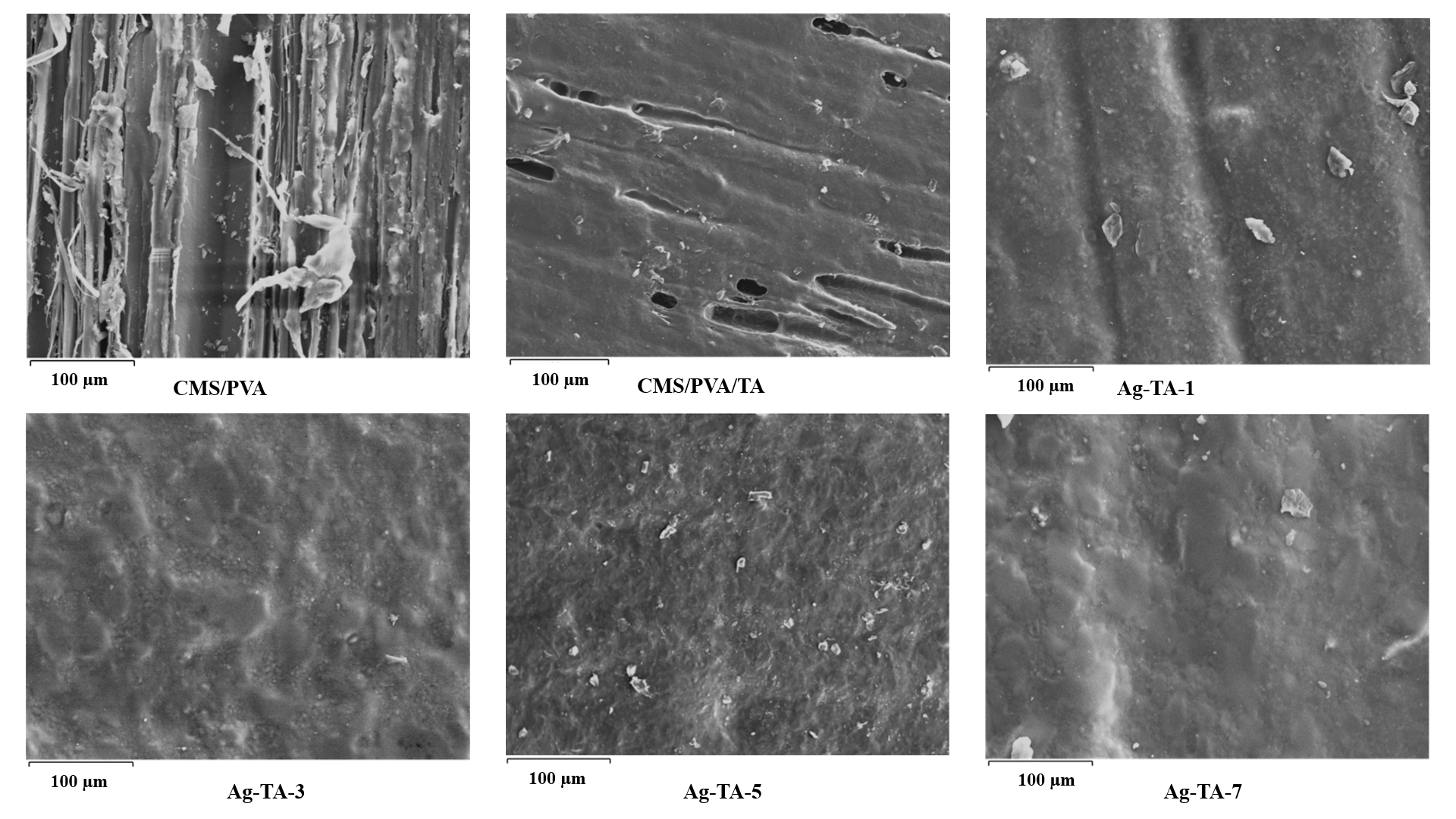


**Figurue S1** SEM images of fracture surface of bio-adhesives after shear strength testing.

**Table S1** Crosslink density of bio-adhesives

| **Samples** | **Crosslink density (g×cm^-3^)** |
| --- | --- |
| CMS/PVA | 22.67±1.51 |
| CMS/PVA/TA | 27.63±1.84 |
| Ag-TA-1 | 45.48±1.51 |
| Ag-TA-7 | 64.09±1.07 |

**Table S2 Cost analysis for manufacturing 1000 mL of Ag-TA-5**

| **Materials** | **Price (USD/Unit)** | **Price for manufacturing** |
| --- | --- | --- |
| CSW (kg) | - | - |
| NaOH (kg) | 2.1 | 0.00015 |
| HCl (L) | 5.4 | 0.16 |
| Chloroacetic acid sodium salt (kg) | 48 | 4.2 |
| Ethanol (L) | 10.2 | 8.55 |
| Isopropanol (L) | 7.1 | 7 |
| TA (kg) | 240 | 12 |
| AgNO_3_ (kg) | 1,650 | 0.19 |
| PVA (kg) | 41 | 2.26 |
| Na_2_CO_3_ (kg) | 30 | 0.33 |
| Deionized water (L) | 0.44 | 0.49 |
| Electricity (unit) | 56 | 3.174 |
| Total | - | 38.35 |
